# Gender-Dependent Associations of Anxiety and Depression Symptoms With Eating Disorder Psychopathology in a Representative Population Sample

**DOI:** 10.3389/fpsyt.2021.645654

**Published:** 2021-02-26

**Authors:** Mareike Ernst, Antonia M. Werner, Ana N. Tibubos, Manfred E. Beutel, Martina de Zwaan, Elmar Brähler

**Affiliations:** ^1^Department of Psychosomatic Medicine and Psychotherapy, University Medical Center of the Johannes Gutenberg-University Mainz, Mainz, Germany; ^2^Department of Psychosomatic Medicine and Psychotherapy, Hannover Medical School, Hanover, Germany

**Keywords:** anxiety, depression, eating disorders, population, gender differences, representative survey

## Abstract

**Background:** Evidence shows that anxiety and depressive disorders play an important role in eating disorder behavior. However, given the epidemiology of eating disorders, there is a need to investigate potentially gender-specific connections.

**Method:** This study tested the associations of anxiety and depression symptoms with eating disorder symptoms and behaviors and explored whether they differed between men and women. Within a population-representative survey (*N* = 2,510; ages 14–94), participants completed measures of depression symptoms (PHQ-2), anxiety symptoms (GAD-2), and eating disorder symptoms (EDE-Q8). We conducted linear regression analyses of the EDE-Q8 sum score and General Linear Models on the three behaviors overeating, binge eating, and compensatory behaviors (self-induced vomiting/use of laxatives/excessive exercising).

**Results:** Depression and anxiety symptoms were related to more eating disorder symptoms in men and women (irrespective of BMI, age, and income). The association of depression and eating disorder symptoms was slightly stronger in women. Overeating was more common in men and in depressed individuals, whereas compensatory behaviors were more common among anxious individuals, especially anxious women.

**Conclusion:** The study extends previous research by using gender-specific methods in a representative sample. It indicates similarities and differences between men and women regarding disordered eating on a population level.

## Introduction

A large body of research has shown deviations from healthy emotional life in individuals with eating disorders. For instance, emotion regulation deficits (e.g., lack of emotional awareness) were found in all diagnosis groups (1). Emotion regulation can be summarized as the capacity to understand one's emotions and to modulate one's emotional response (2). Individuals do this in different ways. For those who do not use adaptive strategies (such as reappraisal), eating disorder behavior can instead serve the purpose to attenuate difficult emotions. For example, Heatherton and Baumeister (3) proposed that binge eating serves as an escape from distressing self-perceptions, thus providing relief.

Indeed, a range of negative subjective states were initiating and/or maintaining factors of different kinds of eating disorder behavior (4). For instance, stress and experimentally induced negative mood increased the likelihood for female, obese study participants to lose control over the amount of food they consumed (5, 6). Likewise, anxiety (comprising symptoms of six anxiety disorders) exacerbated emotional eating and loss of control over eating in adolescents (7). Eating has been shown to alleviate distress in individuals who lacked other, more constructive coping mechanisms to calm themselves (e.g., after experiencing life stressors or experimental manipulations that led to anger or sad mood) (8, 9). Unpleasant emotions, namely sadness, anger, and fear were also related to *restrictive* eating behaviors (10, 11).

More generally, there is a substantial overlap between eating disorders and depressive (12, 13) and anxiety disorders (14, 15). There are different explanatory models for this observation. It has been hypothesized that depression and anxiety disorders are sequelae of eating disorders, or vice versa. Further, eating disorder symptoms could be manifestations of depression and anxiety disorders (depending on an individual's age and gender), or they could share common etiological factors (12, 16). Eating disorder, depression and anxiety symptoms belong to an internalizing phenotype of psychopathology that is much more common in women (17–19). Hence eating disorder research has heavily focused on women and only few studies have investigated large samples of men and women using gender-sensitive or gender-specific methods. However, such approaches are crucial to identify potential targets for prevention and treatment that are relevant in both men *and* in women, or only in men *or* in women [e.g., (20–23)].

The necessity for the investigation of gender-dependent patterns is underscored by previous research that has found different conditions underlying higher bodyweight in men and women. For instance, the association of anxiety and depression symptoms with higher bodyweight was moderated by gender (24) in the sense that women with anxiety and depression symptoms were more likely to have a higher bodyweight than men who reported anxiety and depression symptoms. Likewise, depression was more closely related to obesity in women in the community (25, 26). Similar results were reported by studies that contained measures of eating disorder symptoms or specific behaviors: In path models of binge eating within a sample of undergraduate students, negative affect (comprising e.g., depression symptoms) interacted with loss of dietary restraint in obese women, but not in obese men (27). Based on a large sample of American adults, Cotter and Kelly (28) reported that the association between stress and stress-related eating (eating more than usual as well as eating in order to feel better) was stronger in women. However, there is a lack of research analyzing gender-dependent associations (i.e., by modeling interaction terms) with more cognitive symptoms (including, e.g., preoccupation with food). There is also a research gap with respect to gender-dependent effects of anxiety and depression symptoms on both restrictive eating behaviors as well as compensatory behaviors (such as vomiting, or use of laxatives).

The aim of the present study was to expand previous research by using a large, representative population sample (*N* = 2,510) and by investigating eating disorder psychopathology (including cognitive symptoms such as guilt about eating) as well as actual behaviors of different kinds (overeating, binge eating, and compensatory behaviors). The sample included the entire adult age range, all bodyweight categories, and all income brackets. We investigated the relationships of depression and anxiety symptoms with eating disorder symptoms and behaviors measured by the EDE-Q8, a validated short form of the Eating Disorder Questionnaire (29) and whether these associations differed as a function of gender. In line with previous research cited above, which heavily focused on bodyweight (i.e., obesity), we expected more relevant relationships of the variables of interest within the women in our sample.

In particular, we tested the following hypotheses:

1) a) There are positive associations of depression symptoms and anxiety symptoms with global eating disorder psychopathology in the general population.
b) These associations are modified by gender: they are stronger within women compared to men.

2) a) There are associations of depression symptoms and anxiety symptoms with specific behaviors (overeating, binge eating, compensatory behaviors).
b) These associations are modified by gender, too: they are stronger within women.

## Materials and Methods

### Participants

From September to November 2016, a representative sample of the German population was surveyed by the demographic consulting company USUMA (based in Berlin, Germany). Participants were chosen via a random route procedure. To be included, individuals had to be 14 years of age or older and to have sufficient understanding of the German language. The final sample used in the present investigation was representative of the German population regarding age, gender, and geographic region. Out of 4,902 designated addresses, 2,510 households participated (51.2%). Persons in multi-person households were randomly selected using a Kish-Selection-Grid. All participants provided informed consent. In the case of minors, participants gave informed assent with informed consent being provided by their parents/legal guardians.

Responses were anonymous. Socio-demographic information was obtained in an interview-format by the research staff and all other information was provided via a questionnaire (handed out with by a sealable envelope). Completed questionnaires were linked to respondent's demographic data without containing any identifying information. The study was conducted in accordance with the Declaration of Helsinki, and fulfilled the ethical guidelines of the International Code of Marketing and Social Research Practice of the International Chamber of Commerce and of the European Society of Opinion and Marketing Research. The study was approved by the Ethics Committee of the Medical Department of the University of Leipzig. In total, 2,510 individuals participated (1,171 men and 1,339 women).

### Measures

We used the eight-item version of the Eating Disorder Examination Questionnaire (EDE-Q8) to assess *eating disorder psychopathology*. It has previously shown excellent item characteristics and model fit indices as well as good reliability and convergent validity in large, representative samples of the population (30). In the present sample, internal consistency of the scale was also excellent (ω = 0.92). The eight items cover the topics restraint over eating, food avoidance, preoccupation with food, feelings of fatness, desire to lose weight, guilt about eating, dissatisfaction with weight, and discomfort seeing one's body. With reference to the last 28 days, participants are asked to report how often they were affected by the respective item. Response options range from 0 = never to 6 = every time. Using the same format, three additional items assess the frequency of important *behavioral* aspects of eating disorders. They cover the frequency of overeating, binge eating (i.e., overeating with loss of control while eating), and self-induced vomiting/use of laxatives/excessive physical exercise (in the following summarized as compensatory behavior).

We used the GAD-2 (the two item short form of the Generalized Anxiety Disorder GAD-7) to assess *anxiety symptoms* (31). It starts with the question: “Over the last week, how often have you been bothered by the following problems?” and assesses two symptoms of generalized anxiety disorder: “Feeling nervous, anxious, or on edge,” and “Not being able to stop or control worrying. Response options range from 0 = not at all to 3 = nearly every day, yielding a sum score from 0 to 6. A cut-off score of >=3 has yielded good sensitivity (86%) and specificity (83%) (32). In the present sample, the GAD-2 showed good internal consistency (ω = 0.79).

Following the same instruction question, the PHQ-2 assesses the main criteria of major *depression* according to DSM-5: “Little interest or pleasure in doing things” and “Feeling down, depressed or hopeless” (33). Response options also range from 0 = not at all to 3 = nearly every day and yield a sum score between 0 and 6. A cut-off score of >=3 deemed ideal to identify clinically relevant symptom severity (with a sensitivity of 87% and a specificity of 78%) (33, 34). The PHQ-2 showed good internal consistency in the present sample (ω = 0.81).

We used Body Mass Index (BMI) (kg/m^2^) to control analyses for participants' bodyweight. It was calculated based on their self-reported height and weight.

We calculated equivalised income according to the OECD guideline (35) by dividing the household income through the square root of people in household. The result was then recoded into the following categories: 1 = <1,250€, 2 = 1,250–2,500€, 3 = >2,500€.

Other sociodemographic information such as age and gender were assessed via self-report.

### Statistical Analyses

Sample characteristics are reported as means and standard deviations or percentage and number of cases, respectively.

To investigate research questions (1) (a) and (b), we conducted a hierarchical linear regression analysis of global eating disorder psychopathology (operationalized as the sum score of the EDE-Q8). Hierarchical linear regression allows for testing whether the introduction of new predictors adds to the explanation of the dependent variable's variance in a statistically significant way. Model 1 contained the sociodemographic variables age (as a continuous variable), gender (coded 1= men, 2 = women), and income. In model 2, the (centralized) PHQ-2 and GAD-2 sum scores were added to test specific associations of depression and anxiety symptoms and eating disorder symptoms. In model 3, we added an interaction of each of the two scores with gender to investigate possible gender-dependent differences in the depression and anxiety symptoms' associations with eating disorders. We performed a sensitivity analysis using the calculator provided by Soper (36). We tested the sample size required to observe a small effect (*f*^2^ = 0.02) taking our largest regression model with eight predictors into account. It yielded a minimum sample size of 755, indicating that our investigation had sufficient statistical power. Regression models were checked for multicollinearity using the variance inflation factor (VIF). All VIF scores were below 3 [10 being the critical threshold (37)], indicating no concerning level of multicollinearity (The hierarchical linear regression analysis was followed by two gender-stratified models to gain a better understanding of gender-dependent associations).

To investigate research questions (2) (a) and (b), the association of depression and anxiety symptoms with diagnostically relevant behaviors, we calculated a General Linear Model (GLM) on each of the EDE-Q8's three items assessing the frequency of different behaviors: Overeating, binge eating, and compensatory behaviors (self-induced vomiting, use of laxatives, and excessive physical exercise). The GLMs contained the fixed effects gender, anxiety symptoms, depression symptoms, and the covariates age, income, and BMI. GLMs allow for a robust comparison of mean values (controlling the overall α-level), the modeling of covariates and interaction terms, and the ascertainment of the magnitude of observed effects. We chose separate GLMs as the three variables could not be aggregated in a meaningful way to form a sum score and were only weakly to moderately correlated with each other (overeating and binge eating: *r* =0.533, *p* < 0.001; overeating and compensatory behaviors: *r* = 0.118, *p* < 0.001; binge eating and compensatory behaviors: *r* = 0.255, *p* < 0.001).

*P*-values correspond to two-tailed tests. Analyses were carried out using SPSS 24 for Windows. Regression coefficients and effect sizes are interpreted following Cohen (38).

## Results

### Sample Description

The sample's age ranged from 14 to 94 years with a mean age of 48.4 years (SD = 18.2). It contained slightly more women (*N* = 1,339) than men (*N* = 1,171). The sample was representative for the German population in regard to age and gender. Bivariate correlations showed that female gender was related to reporting more depression symptoms, anxiety symptoms, and global eating disorders symptoms. All of these symptoms were also positively correlated with each other as well as with the reported frequency of the key behaviors overeating, binge eating, and compensatory behaviors (Table 1). Higher age was related to higher income, higher BMI, more depression symptoms and fewer episodes of overeating.

**Table 1:** Means, standard deviations, and correlations among measures.

**Variable**	**Mean (SD)/percentage**	**Gender**	**Income**	**BMI**	**PHQ-2**	**GAD-2**	**EDE-Q8**	**Overeating**	**Binge eating**	**Comp. behaviors**
Age	48.4 (18.2)	0.030	0.245^**^	0.188^**^	0.051^*^	0.002	−0.019	−0.062^*^	−0.014	−0.031
Gender	1_Men_ = 46.65 2_Women_ = 53.35		−0.093^**^	−0.067^**^	0.088^**^	0.115^**^	0.202^**^	−0.097^**^	0.007	0.065^**^
Income	1.55 (0.55)			−0.009	−0.056^**^	−0.062^**^	−0.031	−0.026	−0.040	−0.031
BMI	25.76 (4.68)				0.119^**^	0.072^**^	0.381^**^	0.169^**^	0.182^**^	0.038
PHQ-2	0.69 (1.15)					0.38^**^	0.124^**^	0.101^**^	0.104^**^	0.031
GAD-2	0.69 (0.14)						0.344^**^	0.204^**^	0.232^**^	0.089^**^
EDE-Q8	7.17 (10.19)							0.270^**^	0.332^**^	0.153^**^
Overeating	1.20 (3.41)								0.593^**^	0.118^**^
Binge eating	0.56 (2.17)									0.255^**^
Comp. behaviors	0.08 (0.92)									

*Bivariate analyses are Pearson product-moment correlations, Spearman's Rho for categorical variables*.

* *p ≤ 0.05*,

** * p ≤ 0.01. Statistics of categorical variables indicate percentages. PHQ-2, GAD-2, and EDE-Q8 are reported as the sum score; for the three behaviors, frequency (over the last 28 days) is reported. Comp. behaviors = compensatory behaviors. Income: Equivalised income calculated according to the OECD guideline (35): household income/√(people in household); household income per month: 1 = < 1,250€, 2 = 1,250–2,500€, 3 = >2,500€. N = 2,404*.

### Eating Disorder Symptoms (EDE-Q8 Sum Score)

**Figure 1:**
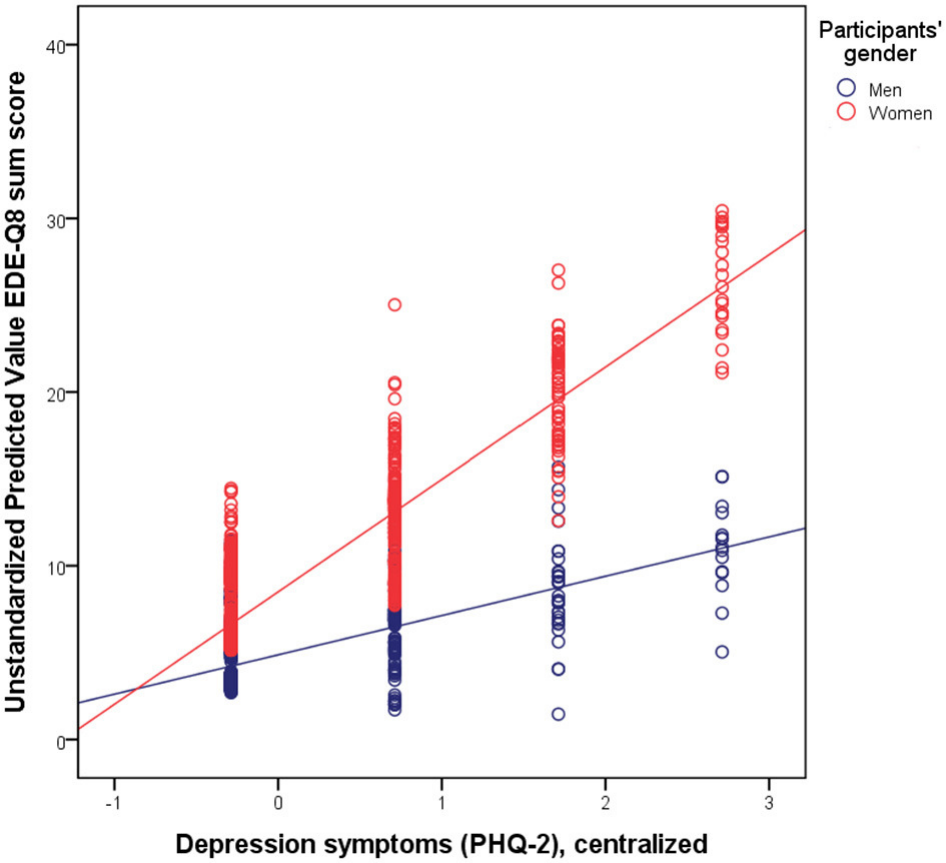
Visualization of the interaction: there was a closer association of eating disorder symptoms as measured by the EDE-Q8 with depression symptoms as measured by the PHQ-2 in women.

In order to investigate our first research question, we conducted a hierarchical linear regression analysis (Table 2). The inclusion of depression symptoms and anxiety symptoms in Model 2 and the inclusion of respective interactions with gender in Model 3 added statistically significant gains in explained variance. The final model explained 30% of the variance in eating disorder symptoms. Positive associations with a higher EDE-Q8 sum score were observed for lower age, female gender, higher BMI, anxiety symptoms, and depression symptoms. The interaction term of depression symptoms and gender was significantly associated with eating disorder symptoms: It indicated that the relation was stronger in women (Figure 1).

**Table 2:** Hierarchical linear regression of eating disorder symptoms (dependent variable: EDE-Q8 sum score).

	**Model 1 (Δ*****R***^****2****^ **=** 0.200**; adj. ***R***^****2****^ **=** 0.199**)			**Model 2 (Δ*****R***^****2****^ **=** 0.099**; adj. ***R***^****2****^ **=** 0.298**)			**Model 3 (Δ*****R***^****2****^ **=** 0.007**; adj. ***R***^****2****^ **=** 0.304**)		
	**β**	**95%CI B (L; U)**	***p***	**β**	**95%CI B (L; U)**	***p***	**β**	**95%CI B (L; U)**	***p***
Gender	0.25	2.09; 2.83	**<0.001**	0.21	1.76; 2.46	**<0.001**	0.21	1.79; 2.48	**<0.001**
Age	−0.10	−0.07; −0.03	**<0.001**	−0.11	−0.08; −0.04	**<0.001**	−0.10	−0.08; −0.04	**<0.001**
Income	0.02	−0.29; 1.10	0.25	0.04	0.08; 1.38	**0.028**	0.39	0.06; 1.35	**0.032**
BMI	0.41	0.80; 0.96	**<0.001**	0.37	0.74; 0.89	**<0.001**	0.37	0.72; 0.87	**<0.001**
Depression symptoms				0.21	0.65; 1.61	**<0.001**	0.21	1.27; 2.21	**<0.001**
Anxiety symptoms				0.13	1.39; 2.32	**<0.001**	0.13	0.58; 1.55	**<0.001**
Depression symptoms × gender							0.08	−0.38; 0.59	**0.004**
Anxiety symptoms × gender							0.01	0.22 1.15	0.66

*Effect coding for gender: men = −1, women = 1. Income: Equivalised income calculated according to the OECD guideline (35): household income/√(people in household); household income per month: 1 = <1,250€, 2 = 1,250–2500€, 3 = >2,500€. Bold values indicate statistically significant predictors*.

For a better understanding of the observed interaction effect, we conducted gender-specific analyses (Table 3). They showed that depression and anxiety symptoms added statistically significant gains in explained variance and were relevant predictors of eating disorder psychopathology. This was true in men and in women (with a larger beta weight for the predictor depression symptoms among women). The relations of younger age with more eating disorder symptoms were only present in women. In women, the model explained a larger proportion of variance (32 vs. 18%).

**Table 3:** Hierarchical linear regression of eating disorder symptoms (dependent variable: EDE-Q8 sum score), stratified by gender.

	**Men**						**Women**					
	**Model 1 (Δ*****R***^****2****^ **=** 0.131**; adj. ***R***^****2****^ **=** 0.128**)			**Model 2 (Δ*****R***^****2****^ **=** 0.057**; adj. ***R***^****2****^ =0.184**)			**Model 1 (Δ*****R***^****2****^ **=** 0.192**; adj. ***R***^****2****^ **=** 0.190**)			**Model 2 (Δ*****R***^****2****^ **=** 0.132**; adj. ***R***^****2****^ **=** 0.321**)		
	**β**	**95%CI B (L; U)**	***p***	**β**	**95%CI B (L; U)**	***p***	**β**	**95%CI B (L; U)**	***p***	**β**	**95%CI B (L; U)**	***p***
Age	0.00	−0.03; 0.03	0.96	−0.01	−0.03; 0.02	0.63	−0.16	−0.13; −0.06	**<0.001**	−0.16	−0.13; −0.07	**<0.001**
Income	0.03	−0.38; 1.26	0.30	0.05	−0.14; 1.45	0.11	0.02	−0.65; 1.53	0.43	0.04	−0.22; 1.78	0.13
BMI	0.36	0.59; 0.82	**<0.001**	0.36	0.60; 0.82	**<0.001**	0.44	0.86; 1.09	**<0.001**	0.38	0.74; 0.95	**<0.001**
Depression symptoms				0.13	0.37; 1.64	**0.002**				0.27	1.75; 3.11	**<0.001**
Anxiety symptoms				0.13	0.36; 1.59	**0.002**				0.12	0.46; 1.84	**0.001**

*Income: Equivalised income calculated according to the OECD guideline (35): household income/√(people in household); household income per month: 1 = <1,250€, 2 = 1,250-2500€, 3 = >2,500€. Bold values indicate statistically significant predictors*.

### Diagnostically Relevant Behaviors

**Figure 2:**
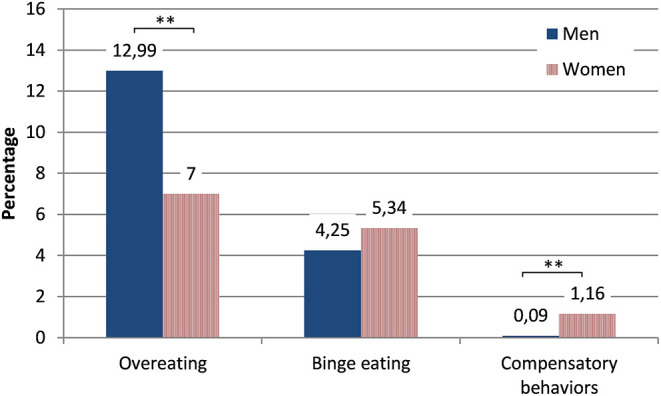
Percentage of individuals of the general population who reported that the respective behavior had occurred at least four times over the course of the last month (stratified by gender). Asterisks indicate statistically significant differences.

Figure 2 depicts the percentage of men and women who reported that the respective behavior occurred at least four times over the course of the last month [the DSM-5 criteria state weekly occurrence (39)]. Overeating was most common, but not all individuals who reported having eaten large amounts of food reported to have felt a loss of control while doing so. Compensatory behaviors (self-induced vomiting, intake of laxatives, or excessive exercise) were the least common behaviors.

The results of the GLMs are shown in Table 4. All three diagnostically relevant behaviors were more common in younger individuals and in individuals with a higher BMI (with small effect sizes for these differences). Overeating was more common in men and compensatory behaviors were more common in women.

**Table 4:** Results of the General linear models of diagnostically relevant behaviors.

**Model**	***F***	***p***	**partial *η^2^***
**1. Dependent variable: Overeating (*R*^2^ = 0.066)**			
Gender	6.23	**0.013**	0.003
Age	19.81	**<0.001**	0.009
BMI	68.38	**<0.001**	0.031
Income	0.08	0.78	0.000
Depression symptoms	15.11	**<0.001**	0.007
Anxiety symptoms	4.25	**0.039**	0.002
Gender x Depression symptoms	0.16	0.694	0.000
Gender x Anxiety symptoms	0.00	0.966	0.000
**2. Dependent variable: Binge eating (*****R***^**2**^ **=** **0.077)**			
Gender	1.40	0.237	0.001
Age	4.71	**0.030**	0.002
BMI	66.29	**<0.001**	0.030
Income	0.40	0.527	0.000
Depression symptoms	39.25	**<0.001**	0.018
Anxiety symptoms	1.55	0.213	0.001
Gender × Depression symptoms	0.93	0.334	0.000
Gender × Anxiety symptoms	0.01	0.934	0.000
**3. Dependent variable: Compensatory behaviors (*****R***^**2**^ **=** **0.018)**			
Gender	9.86	**0.002**	0.005
Age	3.09	**0.079**	0.001
BMI	13.99	**<0.001**	0.007
Income	0.29	0.592	<0.001
Depression symptoms	0.00	0.982	0.000
Anxiety symptoms	6.29	**0.012**	0.002
Gender × Depression symptoms	0.05	0.816	0.000
Gender × anxiety symptoms	3.87	**0.040**	0.002

*Income: Equivalised income calculated according to the OECD guideline (35): household income/√(people in household); household income per month: 1 = <1,250€, 2 = 1,250-2500€, 3 = >2,500€. Bold values indicate statistically significant effects*.

There was no association of gender with binge eating. Regarding depression and anxiety symptoms, we observed different patterns of relations with the three different behaviors: Overeating was more common in those with more anxiety symptoms and binge eating was more common in participants with more depression symptoms. Self-induced vomiting and other compensatory behaviors were more common among those with more severe anxiety. There was also an interaction, indicating that the latter association was stronger in women. All effects observed within the GLMs were small (η^2^ <0.06).

## Discussion

We used a large sample representative of the German population to explore the relations of eating disorder symptoms as well as specific behaviors with depression and anxiety symptoms in men and women. Global eating disorder symptoms (which included cognitive symptoms such as preoccupation with food and self-evaluations such as dissatisfaction with weight) were more common in women and in individuals with higher bodyweight. These findings corroborate previous research (23, 30, 40), including investigations that used the longer version of the EDE-Q in large population samples in other countries (41). Regarding the different behaviors, our results are also in line with previous epidemiological investigations which found that overeating was the most common type of disturbed eating in the general population. Previous studies also found that men were more likely to indicate overeating, however, more women reported binge eating (42, 43). The finding that compensatory behaviors were more common in women mirrors previous reports of higher prevalence rates of, for example, bulimia in women (18).

### Evidence for the Association of Depression and Anxiety Symptoms With Global Eating Disorder Psychopathology and Differences Between Men and Women

The present study expands previous research by showing that global eating psychopathology was associated with depression symptoms (anhedonia and depressed mood) and anxiety symptoms (nervousness, not being able to stop/control worrying) in the German general population irrespective of participants' bodyweight, age, and income. The associations of eating disorder psychopathology and behaviors with anxiety and depression symptoms are in line with previous reports about psychological comorbidities among those with eating disorders (50% with anxiety disorders and 40% with mood disorders) (18). In our study, anxiety was also more closely related to eating disorder symptoms and behaviors than depression. Whilst we found that depression symptoms statistically predicted eating disorder symptoms in men and women, this association was comparatively stronger among women.

It has been proposed that negative, self-evaluating cognitions constitute the main overlap between depressive disorders and eating disorders (44, 45). Along these lines, previous research has stressed the relevance of sociocultural risk factors such as female gender role socialization in explaining the gender gap in eating disorders (46). As one's physical attractiveness, including the shape and weight of the body (which is judged against a thin ideal) is more relevant for women's social evaluation than men's (47), these aspects might be more closely related to negative affect in women (48, 49). Striving for thinness by the means of dieting, exercise, and other weight control behaviors might give the individual a sense of being in control, e. g. after a recent stressful life event (50). Moreover, there are other aspects of eating disorders that play a particularly important role for women that are not related to attractiveness. Among them are questions of self-determination, agency, and feminine identity (51) as well as problematic mother-daughter relationships (52).

The fact that the gender-specific regression model including anxiety and depression symptoms explained more variance in the female sample might indicate that the model lacked factors that are more relevant for men's eating psychopathology. Previous research has suggested that those might be weight history (20) and aspects of athletic achievement (53). Furthermore, the symptoms assessed by the EDE-Q8 comprise feelings of fatness and the desire to lose weight as opposed to, for example, muscularity-oriented diet and exercise and muscularity-oriented dissatisfaction. It thus omits concerns that were especially relevant for men with eating disorders (54–56).

### Evidence for Associations of Depression and Anxiety Symptoms With Diagnostically Relevant Behaviors

In line with previous research, we observed associations of depression and anxiety symptoms with actual disturbed eating behavior which comprised overeating, binge eating, and compensatory behaviors. In our study, depression symptoms were especially common among individuals who reported binge eating. This corresponds to the substantial overlap of depression and binge eating disorder which is characterized not only by eating large portions, but also by a loss of control (57, 58). Congruently, negative affect, maladaptive cognitions, and inadequate coping strategies were related to its onset as well as to its maintenance (59). Depression symptoms were also related to worse treatment outcomes in overweight individuals who tried to lose weight (57).

Regarding the frequency of overeating episodes, a small association with anxiety was present alongside the association with depression symptoms. Previous research has found that overeating might provide relief or a feeling of calm (7, 8). However, overeating improved participants' mood only for short time periods (9).

More anxiety symptoms (but not more depression symptoms) were observed among those reporting compensatory behaviors. This is in line with previous research that had investigated negative and positive affect as predictors of vomiting (60). We also observed an interaction of gender and anxiety symptom burden, indicating that anxious women might be at a particularly high risk to engage in these behaviors. Previous research has indicated that negative affect might trigger compensatory behaviors in women, but not in men (43). This interaction effect was the only one we observed; gender did not moderate the associations of depression and anxiety symptoms and overeating and binge eating, respectively. Thus, these analyses do not contribute to a better understanding of the univariate gender difference we observed with respect to the prevalence of overeating in the population.

However, all observed effects were small. This might be due to the sample (as most of the participants within the population survey reported neither eating disorder symptoms nor anxiety/depression symptoms). Also, as the present sample was representative of the population, participants' mean age was considerably higher than in many clinical investigations. Research has shown that the prevalence of eating disorders declines with age (61). Lastly, newer, more fine-grained methods such as Ambulatory Assessment give a better insight into chronological sequences of feelings and behaviors than questionnaire surveys capturing the last weeks.

## Strengths and Limitations

Strengths are the large, representative sample (including individuals with and without eating disorder, depression, and anxiety symptoms), the broad age range, and the inclusion of all bodyweight categories. To our knowledge, it is the first population-representative, gender-specific investigation of the association of depression and anxiety symptoms with self-reported eating disorder symptoms comprising both cognitive symptoms (such as preoccupation with food) and behaviors (such as vomiting).

However, the results must also be judged against the backdrop of the study's limitations. First, the present investigation is a cross-sectional analysis of survey data. It does not allow for inferences with respect to cause and effect. BMI was assessed via self-report. Diagnostically relevant behaviors were assessed in terms of their frequency over the last month and not as “at least weekly” episodes in line with the DSM-5 criteria. The present study did not contain sufficient data to diagnose eating disorders. We did not inquire whether participants had ever received diagnoses of eating disorders either.

Further, given the fact that we used a community sample, some proportions were quite small (e.g., individuals reporting compensatory behaviors). An investigation of the study's research questions within large patient samples or high-risk populations (e.g., athletes) will help to ascertain the clinical relevance of our findings. It is another limitation that the behaviors were assessed using single items and that all questionnaires were screening measures (as the format of the large survey study did not allow for the inclusion of more time-consuming instruments). As a result, we can only report associations of eating disorder symptoms and behaviors with the symptoms contained in the short screeners, but not with other symptoms also present in the full manifestation of the disorders such as e. g. fatigue/loss of energy in the case of depression. Also, the GAD-2 only covers two main symptoms of generalized anxiety disorder. Our survey did not include measures of, for example, social anxiety disorder which has also been found to overlap with eating disorders [e.g., (62)]. This limitation might also have contributed to the relatively small proportions of explained variance within the different statistical models.

As noted above, many conceptions of eating disorder symptomatology (including the EDE-Q8) do not include specific features of men's eating disorders. As a consequence, currently used questionnaires might not be as sensitive to eating disorder symptomatology in men as in women and the present study might have underestimated men's symptom burden. Studies investigating differences between men and women will also benefit from collecting information such as the perception of gender roles, gender role self-concept, and gender role conflict. What is more, sexual orientation plays an important role as both non-heterosexual men and women were more vulnerable to body weight dissatisfaction (63). The present research did not include such measures which is why they could not be probed as potential risk factors. Moreover, gender was only assessed in a binary way (women/men).

Lastly, all reported effects observed within the General Linear Models were small and none of them explained more than 8% of the respective criterion's variance. Smaller proportions of explained variance are common in epidemiological research, large surveys, and other fields with greater amounts of unexplainable variation than in controlled experimental environments.

## Conclusion

The present results from a representative sample of the German population show that depression as well as anxiety symptoms were related to eating disorder symptoms (comprising restraint, eating concern, weight concern, and shape concern) in men and women irrespective of their age, BMI, and income. Overeating was the most common behavior. It was more common among men than among women, but similar numbers of men and women reported binge eating episodes. Women were at a higher risk for global eating disorder symptom burden and compensatory behaviors (self-induced vomiting, use of laxatives, and/or excessive exercise), although the latter behaviors were comparatively rare.

Our results indicate that specific behaviors observed in individuals with eating disturbances might be related to depression and anxiety symptoms. Overeating and binge eating might be relatively common among those coping with depression, while resorting to—in some instances self-damaging—compensatory measures might primarily serve to diminish anxiety/nervousness (e.g., regarding weight gain). The latter might be more common among women as women face greater societal pressure to be thin. Thus, individuals suffering from different eating disorder symptoms could benefit from learning strategies to cope with different kinds of symptoms, for example, nervousness or depressed mood.

As the observed gender-dependent effects were small, the study suggests some degree of clinical similarities between women and men with eating disorder symptoms. Going forward, the conceptualization of eating disorder psychopathology as a predominantly young, female problem should thus be superseded by more inclusive and differentiated approaches. Furthermore, the fact that we found the reported relations on a population level might indicate targets for prevention. Members of the general public who do not suffer from clinically relevant symptoms or who do not (yet) fall into extreme bodyweight categories might also benefit from guidance in dealing with negative emotions in daily life.

## Data Availability Statement

The raw data supporting the conclusions of this article will be made available by the authors, without undue reservation.

## Ethics Statement

The studies involving human participants were reviewed and approved by Ethics Committee of the Medical Department of the University of Leipzig. All participants provided informed consent. In the case of minors, participants gave informed assent with informed consent being provided by their parents/legal guardians.

## Author Contributions

ME, AW, and AT designed the study. EB and MdZ were involved in data acquisition. ME and AW drafted the manuscript. ME conducted the statistical analyses and produced the figures. AT, MB, MdZ, and EB revised the manuscript for important intellectual content. All authors were involved in the interpretation of the results, responsible for all aspects of the work, and have approved the final version of the manuscript.

## Conflict of Interest

The authors declare that the research was conducted in the absence of any commercial or financial relationships that could be construed as a potential conflict of interest.
